# Distance education of midwifery students during the COVID-19 pandemic: Challenges and recommendations

**DOI:** 10.18332/ejm/138594

**Published:** 2021-07-12

**Authors:** Denız Akyıldız

**Affiliations:** 1 Midwifery Department, Faculty of Health Science, Kahramanmaraş Sütçü İmam University, Kahramanmaras, Turkey

**Keywords:** COVID-19, pandemic, student, midwifery, distance education

**Dear Editor**,

COVID-19, starting in December 2019, spread all over the world and was declared as a pandemic and changed a large part of daily life^1,2^. Following the rapid spread of the disease in many countries, measures were taken to prevent contamination and distance education was initiated at universities^3^. COVID-19 pandemic caused the most significant negative effect on education in recent history, affecting large numbers of students in many countries. The distance education in the pandemic caused difficulties, especially in departments that require a clinical learning environment such as midwifery^4,5^. Midwives have an important role in decreasing maternal and neonatal mortality rates, which are the leading development indicators of countries^6,7^. In this respect, the quality of midwifery education is very important.

The transformation of face-to-face education into distance education after the pandemic brought many challenges with it. Midwifery students in low-income households around the world, without internet access and computer, could not benefit from distance education sufficiently. The fact that students are not obliged to attend classes in distance education reduces students’ active participation in the course^8^. Also, the lack of clinical practice and laboratory applications can be regarded as another problem of distance education. To graduate from the midwifery program, students have to meet accreditation standards all over the world. These standards require that students meet a minimum number of clinical practice experiences, including 100 antenatal and 100 postnatal period of care, 40 labour and birth experiences, and 100 neonatal assessments^9^. In the pandemic, the students had difficulties in completing these standards as they were not able to do their clinical internship. Midwifery students experienced difficulties in developing their knowledge and skills due to a lack of internship and laboratory practices. On the other hand, when students go on clinical internships during the pandemic, problems such as students infecting themselves and carrying the infection among patients can occur. Another problem is the need for personal protective equipment for students because social distancing is essentially not possible in midwifery work^3^.

For all these reasons, new strategies should be developed to continue the education of midwifery students to be employed in the future. Higher education institutions are responsible for evaluating both institutional and epidemiological conditions in order to define pathways for graduation courses in midwifery^10^. Contrary to standard education planning, it is crucial to make special education and graduation planning for midwifery students. Course participation can be increased by providing students with internet and computer support, and making it compulsory to attend courses. In addition, it can be ensured that students pass the courses that include laboratory and practice, on the condition that they have to follow these courses after the pandemic. Students who complete their education in the pandemic should work as interns for a period in hospitals. During the pandemic, there is a need for standardized regulation proposals from midwifery associations and organizations regarding midwifery student education.
